# Blenderized tube feeding for children: an integrative review

**DOI:** 10.1590/1984-0462/2022/40/2020419

**Published:** 2021-09-01

**Authors:** Érica Patrícia Cunha Rosa Schmitz, Eliziane Costa da Silva, Ozeas de Lima Lins, Margarida Maria de Castro Antunes, Kátia Galeão Brandt

**Affiliations:** a Universidade Federal de Pernambuco, Recife, PE, Brazil.; b Universidade de Pernambuco, Recife, PE, Brazil.

**Keywords:** Food, formulated, Child nutrition, Enteral nutrition, Patient satisfaction, Child health, Alimentos formulados, Nutrição da criança, Nutrição enteral, Satisfação do paciente, Saúde da criança

## Abstract

**Objective:**

To analyze scientific evidence on the use of blenderized tube feeding in children regarding nutritional composition, family satisfaction, and health outcomes.

**Data source:**

Survey was conducted in the PubMed, Scopus, Embase, and Virtual Health Library (VHL) databases using the following search terms: *blenderized tube feeding* OR *blended tube feeding* OR *homemade* OR *pureed* AND *enteral nutrition* AND *enteral tube*. The methodological quality of the selected articles was evaluated using the Critical Appraisal Skill Programme and Hierarchical Classification of Evidence.

**Data synthesis:**

After analysis, 11 articles were included in the present review. Most studies demonstrated improvements in health outcomes and greater family satisfaction after replacing the commercial enteral feeding with blenderized tube feeding.

**Conclusions:**

When guided and monitored by the healthcare team, a blenderized tube feeding ensures an adequate nutritional composition. The use of this method is also associated with positive health outcomes such as reductions in gastrointestinal symptoms and hospitalizations. Moreover, a high frequency of family satisfaction was verified.%

## RESUMO

**Objetivo:**

Analisar as evidências científicas sobre o uso da dieta enteral artesanal em crianças, no que se refere a composição nutricional, satisfação familiar e desfechos em saúde.

**Fontes de dados:**

Foi realizado levantamento nas bases de dados PubMed, Scopus, Embase e Biblioteca Virtual em Saúde, utilizando a estratégia de busca *blenderized tube feeding* OR *blended tube feeding* OR *homemade* OR *pureed* AND *enteral nutrition* AND *enteral tube*. Os artigos selecionados foram avaliados quanto à qualidade metodológica por meio de dois instrumentos: *Critical Appraisal Skills Programme* e classificação hierárquica das evidências.

**Síntese dos dados:**

Após análise, 11 artigos foram incluídos na revisão. A maioria dos estudos demonstrou melhora dos desfechos em saúde e maior satisfação familiar após a substituição da dieta enteral industrializada pela dieta enteral artesanal.

**Conclusões:**

Observou-se que foi possível garantir uma composição nutricional adequada na dieta enteral artesanal quando orientada e monitorada pela equipe de saúde. Adicionalmente, o seu uso esteve relacionado a desfechos positivos em saúde, como redução das internações hospitalares e redução dos sintomas gastrointestinais. A frequência de satisfação familiar foi elevada.%

## INTRODUCTION

Children with difficulties to maintain adequate nutrition orally may need to have a diet through an alternative feeding route, provided by a tube or gastrostomy for food, called enteral nutrition.^1^ Providing an enteral feeding that is nutritionally complete is extremely important for the child, as it will allow for better growth and development in addition to reducing the chances of developing diseases.^2^

In the past, the blended homemade nutrition, nowadays called blenderized tube feeding (BTF), was the only form of food available for patients unable to eat orally. However, in the mid-20^th^ century, commercial enteral feeding (CEF) was developed and propagated as more efficient and safer for having greater nutritional accuracy, ease of use, and sterility.^3^ This fact made CEF a priority, and BTF started being only used by socially-vulnerable patients, who had limited access to commercial feedings due to their cost.^4^

Recently, a cultural shift toward greater consumption of unprocessed foods, with minimal addition of sugars and preservatives, has also involved caregivers of children fed by probes, arousing new interest in the use of BTF.^5^ ^,^ ^6^ Accordingly, commercial enteral feedings that contained “real foods” in its list of ingredients were also developed such as chicken meat, carrots, peas, beans, among others.

Currently, BTF has been partially or totally used for the nutrition of children in several countries; however, there are still doubts about nutritional safety and the potential benefits of its use. Therefore, this study aimed to analyze the scientific publications existing to date on the nutritional composition of BTF used with children, family satisfaction with its use, and the health outcomes of children who partially or totally use BTF.

## METHOD

This is an integrative literature review, which sought to synthesize the results on the use of BTF by children. To do so, six steps were followed: 1) identification of the problem, with definition of the research question; 2) searching databases and virtual libraries using descriptors; 3) screening and identification of relevant studies; 4) individual reading of the complete texts; 5) data collection, tabulation of studies, and analysis of those that were included; and 6) presentation of results and discussion.^7^

In the first step, the following guiding question was considered: in children who depend on enteral nutrition, how does BTF compare to commercial feedings in terms of nutritional composition, family satisfaction, and health outcomes?

In the second step, the search for references was carried out in the Virtual Health Library (VHL), United States National Library of Medicine (PubMed), Scopus, and Embase databases between June and July 2020, with no year limit as for publication.

The selection of descriptors was guided by their proximity to the object in question, which were all grouped using the Boolean expressions AND and OR: *blenderized tube feeding* OR *blended tube feeding* OR *homemade* OR *pureed* AND *enteral nutrition* AND *enteral tube*.

The search for the articles was independently carried out by two researchers based on selected descriptors, guided by the following inclusion criteria: original articles written in Portuguese, English, and Spanish languages, covering the main theme and addressing the research question. Studies exclusively involving older people and/or adults, who analyzed letters to the editor, duplicates, review-like studies, dissertations, theses, opinion articles, comments, essays, preliminary notes, and manuals were excluded.

In addition, a manual search of reference lists from previous reviews^8^ ^–^ ^11^ was conducted to identify studies that might not have been retrieved by automated search. When it was not possible to obtain complete articles, the authors of such studies were contacted.

Then, after removing duplicates and screening the selected articles, the articles were individually read for data collection and tabulation of studies. After defining the final number of articles to compose the review, an analysis of the methodological quality of the included studies was performed regarding their adherence to the object of this research.

To assess the methodological quality of the included articles, two instruments that enabled the evaluation of different study designs were used: one adapted and translated into Portuguese^12^ ^,^ ^13^ based on the Critical Appraisal Skills Programme (CASP)^14^ and the Agency for Healthcare and Research and Quality (AHRQ),^15^ which classifies studies into seven levels according to the level of evidence.

In the present review, an instrument adapted from CASP was used, which included 10 items to be scored, including: 1) objective; 2) adequate methodology; 3) presentation of theoretical and methodological procedures; 4) adequate sample selection; 5) detailed data collection; 6) relationship between researcher and respondents; 7) respect for ethical aspects; 8) rigor in data analysis; 9) presentation and discussion of results; and 10) research contributions and limitations. Each item was assigned a value of 0 (zero) when the answer was negative, or 1 (one) when the answer to the item was affirmative. The final result was the sum of the scores, whose maximum score was 10 points. At the end of the instrument, the study was classified as level A – 6 to 10 points (good methodological quality and reduced bias) – or level B – up to 5 points (satisfactory methodological quality, but increased risk of bias).

In order to minimize biases, the search, evaluation, and selection of studies were independently carried out by two reviewers. When there was disagreement, a third reviewer was consulted and, finally, there was a consensus discussion on the articles to be included in the review.

## RESULTS

**Figure 1:**
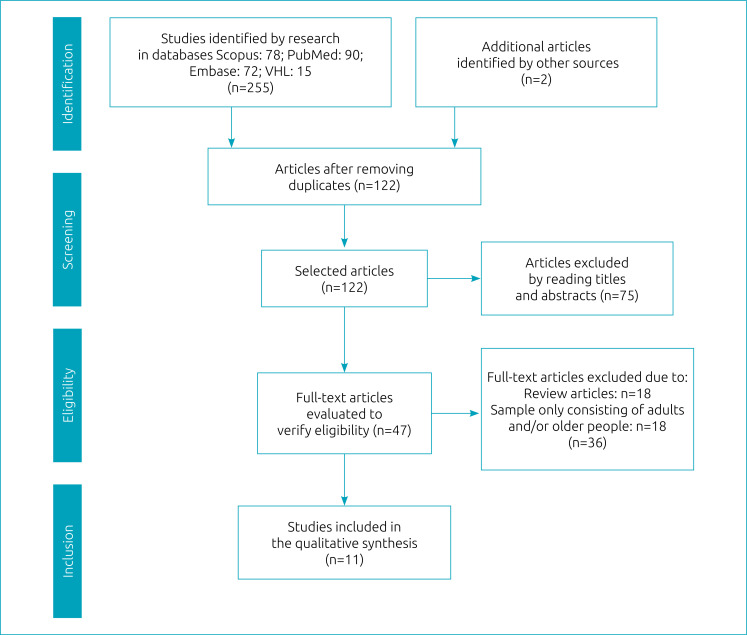
Flowchart of the study search and selection process.

A total of 255 titles were identified. The database with the largest number of articles was PubMed (90), followed by Scopus (78), Embase (72), and VHL (15). Two studies^16^ ^,^ ^17^ were added by searching other sources, such as reference lists in previous reviews.^8^ ^–^ ^11^ Initially, 135 studies were excluded for being duplicates. Then, 75 articles were excluded after reading titles and abstracts because they did not address the research question. 47 articles remained to be read in full. Of these, 36 publications were excluded because they did not meet the inclusion criteria, which resulted in 11 articles in the final sample. Figure 1 presents the study selection strategy flowchart according to the standards of the Preferred Reporting Items for Systematic Reviews and Meta-Analyses (PRISMA).^18^ The main information from the selected studies is shown in Table 1.

**Table 1:** Compiled description and levels of evidence, according to and adapted from the Critical Appraisal Skills Program, and Agency for Healthcare and Research and Quality.

Author, year	Sample	Main findings	CASP AHRQ
Batsis et al., 2020	23 children aged 1 to 18 years	BTF was well tolerated. Gastrointestinal symptoms, such as gagging, nausea, and vomiting, improved in most children.	A VI
Trollip et al., 2020	12 children aged 1 to 14 years	Improvements in overall health, emotional and social well-being, and gastrointestinal symptoms after using the BTF.	A VI
Hron et al., 2019	70 children aged 1 to 18 years	Better clinical results in children who were offered blenderized or commercial feeding with real foods. The total number of emergency visits, hospital admissions, and admissions due to respiratory complications was reduced.	A IV
McClanahan et al., 2019	10 children aged 2 to 8 years	PBEN was well tolerated, with improvement in the observed symptoms, and can improve the health of microbiota in children with chronic diseases using an alternative feeding route.	A IV
Gallagher et al., 2018	20 children aged 1 to 16 years	Participants needed 50% more calories to maintain their BMI while using the BTF. Bacterial diversity and richness in stool samples significantly increased.	A IV
Johnson et al., 2018	433 parents of children fed by probe	Reasons for using a blenderized tube feeding and/or commercial enteral feeding with real foods included the desire to offer whole foods, having family meals, or the fact that they did not like the commercial feeding.	A VI
Orel et al., 2018	37 participants aged 2 to 26 years	After six months of intervention, the Z-scores for weight-for-age and BMI, as well as the percentages of fat and lean mass, were higher in the commercial feeding group than in the blenderized feeding group.	A IV
Epp et al., 201**7**	125 children with mean age of 5.4 years	Of the patients, 89.6% used a blenderized feeding for an average of 71% of the total daily nutrition intake; 83% reported that the diet represented > 50% of their energy requirements.	A VI
Samela et al., 2016	10 children aged 1 to 6 years	90% of children were successful in the shift to a diet formulated with real foods. There was also an improvement in their feces patterns.	A IV
Klek et al., 2014	456 children; 142 of whom with a mean age of 8.7 years	CEF allowed for weight gain and reduced the incidence of infectious complications, the number and length of hospital stays.	A IV
Pentiuk et al., 2011	33 children with a mean age of 34.2 months	52% of children reported a reduction in gagging and vomiting, and 57% of children reported an increase in oral intake after the introduction of BTF.	A IV

BMI: body mass index; PBEN: plant-based enteral nutrition; BTF: blenderized tube feeding; CEF: commercial enteral feeding; CASP: Critical Appraisal Skills Programme; AHRQ: Agency for Healthcare and Research and Quality.

As for language, all articles were published in English and in international journals. Concerning the years of publication, most articles were from 2018 (27.2%), 2019 (18.2%), and 2020 (18.2%), followed by 2017, 2016, 2014, and 2011, each with only one article (9.1%). The study locations were the United States of America (63.6%), Australia (9.1%), Canada (9.1%), Slovenia (9.1%), and Poland (9.1%).

Regarding the sample size, the surveys with the lowest number of participants were those conducted by Samela et al.^19^ and McClanahan et al.,^17^ with 10 children each. The study with the highest number of participants was the one performed by Klek et al.,^20^ which included 456 participants.

Regarding the main findings of the 11 included studies, in nine (81.8%) the BTF or commercial enteral feeding with real foods showed superior results in relation to CEF such as improvement in gastrointestinal symptoms, weight gain, reduction in hospitalizations, and increased family satisfaction. In only two (18.2%) studies CEF showed better results than BTF such as improved nutritional status and reduced hospital admissions and infectious complications in children.

After reading the studies in full, all of them were classified as level A, according to the adapted CASP. According to the AHRQ, four articles (36.4%) were classified as level VI of evidence for being cross-sectional studies, and seven (63.6%), as level IV for being cohorts.

Table 2 shows the percentage distribution of enteral diets provided in the studies. BTF was adopted in nine (81.8%) of them; the commercial enteral feeding with real foods, in six (54.5%); the commercial enteral feeding, in four (36.4%); and the mixed diet (prepared with commercial feeding associated with blenderized feeding, or commercial enteral feeding with real foods associated with blenderized feeding), in five (45.4%).

**Table 2:** Types of enteral nutrition provided to study participants.

Author, year	Blenderized tube feeding n (%)	Commercial enteral feeding with real foods n (%)	Commercial enteral feeding n (%)	Mixed diet* n (%)
Batsis et al., 2020	15 (65.2)	4 (17.4)	––––	4 (17.4)^b^
Trollip et al., 2020	4 (33.3)	––––	––––	8 (66.7)^a^
Hron et al., 2019	11 (15.7)	14 (20.0)	28 (40.0)	17 (24.3)^a^
McClanahan et al., 2019	––––	10 (100.0)	––––	––––
Gallagher et al., 2018	20 (100.0)	––––	––––	––––
Johnson et al., 2018	134 (31.2)	20 (4.6)	213 (49.5)	59 (13.7)^b^
Orel et al., 2018	20 (54.0)	––––	17 (46.0)	––––
Epp et al., 2017	84 (75.0)	1 (<1.0)	––––	27 (24.0)^b^
Samela et al., 2016	––––	10 (100.0)	––––	––––
Klek et al., 2014^3^	456 (100.0)	––––	456 (100.0)	––––
Pentiuk et al., 2011	33 (100.0)	––––	––––	––––

* Commercial+blenderized feeding or blenderized+commercial feeding with real foods;

a commercial+blenderized feeding;

b blenderized+commercial feeding with real foods;

3 all participants initially used the blenderized tube feeding and later shifted to the commercial enteral feeding.

## DISCUSSION

Only two studies^21^ ^,^ ^22^ investigated the nutritional composition of BTF used with children. In the study conducted by Gallagher et al.^21^, for four weeks, 20 children fed by gastrostomy shifted from the use of CEF to BTF. At the beginning of the research, their families received guidance from the nutritionist working at the service and written information for the preparation and provision of BTF. Prescriptions were determined by the nutritionist taking into account the patient's intake at the beginning of the research and the estimated needs. Children were prospectively followed up for six months. Regarding the caloric intake of the diets, it was observed that, in order to maintain a stable body mass index (BMI), the intake through BTF should be 50% higher than that provided to children when having CEF.

The authors^21^ also reported that there is no clarity about the reasons for this greater caloric need when children are having BTF; however, they point out as possible explanations differences in the thermogenic effect of the diets and changes in the digestion and absorption of food, secondary to alteration of the gut microbiota due to the diet. As for macronutrients, it was observed that protein intake was higher in children who were offered BTF; nevertheless, it remained in line with the recommendation of the acceptable macronutrient distribution range (AMDR).

In the observational study conducted by Hron et al.,^22^ children using CEF or BTF (those who were offered 50% or more of homemade feeding or commercial feeding with real foods) were compared, previously followed up at the outpatient clinic of a reference hospital in Boston, USA. It was observed that the caloric intake and the distribution of macronutrients did not differ between the two groups. Considering these findings, the authors emphasize that a nutritionally complete diet is clearly possible to be provided through BTF.

Regarding the content of micronutrients, in both studies,^21^ ^,^ ^22^ the vitamin D content in BTF was lower than the reference dietary intake. The authors recommended for an adequate intake to be guaranteed or that additional vitamin D supplementation to be indicated for preventing deficiency of this micronutrient.

Fiber intake was significantly higher in the group of children who received BTF in the study carried out by Hron et al.,^22^ which was explained by the fact that this food component is reduced or absent in many CEF. On the other hand, it is worth noting the high fiber content of the blenderized tube feeding, due to the risk of obstruction of the probe.^23^

Health outcomes with the use of BTF or commercial feeding with “real foods” were addressed by 10 studies.^16^ ^,^ ^17^ ^,^ ^19^ ^–^ ^22^ ^,^ ^24^ ^–^ ^27^ Of these, eight^17^ ^,^ ^19^ ^,^ ^21^ ^,^ ^22^ ^,^ ^24^ ^–^ ^27^ highlighted positive and superior results of BTF in relation to CEF. Only two studies^16^ ^,^ ^20^ observed better health outcomes after starting the use of CEF.

As for the positive health outcomes after adopting the BTF, in the qualitative study by Trollip et al.^25^, parents reported that after partially introducing BTF there was an improvement in overall health, less propensity to viral diseases, and improvement in children's immunity.

When comparing the clinical outcomes of two groups of children who were offered BTF or CEF, Hron et al.^22^ observed that the annual frequency of emergency visits, the total number of hospital admissions, and hospital admissions due to respiratory problems were significantly lower in the group that used BTF. The authors conclude that BTF is a well-tolerated, safe, and relatively low-cost intervention to improve children's health outcomes.

The impact of introducing food (total BTF or associated with CEF) on the diet of children fed by probe, in relation to the occurrence of gastrointestinal symptoms, was evaluated in six studies.^19^ ^,^ ^21^ ^,^ ^22^ ^,^ ^24^ ^,^ ^25^ ^,^ ^27^ All studies observed a beneficial impact on the introduction of food associated with the reduction of nausea, vomiting, diarrhea,^19^ ^,^ ^22^ ^,^ ^24^ ^,^ ^25^ ^,^ ^27^ gagging,^21^ ^,^ ^24^ reflux, constipation,^25^ abdominal pain, and better tolerance to the diet volume.^22^

The reduction in gastrointestinal symptoms related to food introduction is explained by the authors as being associated with some factors such as slower gastric emptying due to the higher viscosity of the feeding. The higher fiber content in the diets could justify the lower occurrence of diarrhea, abdominal pain, and constipation.^22^ Another hypothesis is that, with whole foods, the chyme would reach the small intestine at a rate that would stimulate a more regular hormonal response, promoting a more physiological motility and reducing gastrointestinal symptoms.^24^

As for anthropometric parameters, three surveys^24^ ^–^ ^26^ reported that children reached growth goals more frequently when they totally or partially used BTF. Batsis et al.^24^ found a significant improvement in height Z-scores after the shift to BTF (total or associated with commercial enteral feeding with real foods). Despite the nutritional guidelines provided by an experienced pediatric nutritionist and aimed at children, the authors emphasize that the significance of the result is unclear, considering the retrospective nature of the study and the challenges of nutritional assessment of children with neurological impairment. It was not possible to confirm the findings of the other two studies^25^ ^,^ ^26^ due to methodological issues of these investigations, as parents or caregivers recorded the children's anthropometric parameters in questionnaires previously sent, which were not directly measured by the researchers. Thus, to confirm the findings, anthropometric measurements and their interpretation require trained healthcare professionals.

It was also possible to demonstrate an adequate weight gain using the blenderized tube feeding. In the study by Samela et al.^19^ who analyzed the use of a commercial enteral feeding containing real foods, all children maintained adequate weight gain for their age at six months and one year of follow-up. The results of Epp et al.^28^ showed that weight loss was less likely in children totally or partially using BTF than in those using CEF; however, they stressed that, in order to maintain adequate weight gain using the BTF, it is vital for patients to carry out a strict follow-up with a nutritionist. Pentiuk et al.^27^ found an average weight gain of 6.2 g per day in children after fundoplication surgery, with the use of planned BTF in order to meet their nutritional needs. This result is justified by individualized nutritional follow-up, with modification of the caloric content of the diet when necessary.

Regarding the benefits for the gastrointestinal microbiota, Gallagher et al.^21^ observed an increase in bacterial diversity and richness in stool samples six months after shifting to BTF. Another observed benefit was the significant reduction of proteobacteria in the patients’ feces when comparing samples before and after six months of using the BTF. Similar findings were obtained in a small pilot cohort^17^, in which, two months after shifting to the commercial enteral feeding with real and organic foods, there were changes in the diversity and abundance of bacterial metabolites. Nevertheless, the authors stressed that the findings did not allow confirming the causal effect of the diet on the improvement in the microbiota profile due to problems such as lack of statistical significance, small number of participants, lack of a control group, or the need for another type of study to confirm the results.

Contrary to previous studies, two studies^16^ ^,^ ^20^ related an improvement in health outcomes after interrupting the BTF and the beginning of the use of CEF. A multicenter observational study^20^ demonstrated that, 12 months after shifting to CEF, children showed greater weight gain, reduced incidence of complications from infectious diseases and the number and length of hospital stays. These changes have significantly reduced the average annual costs of hospitalization. The good results were attributed to the fact that the CEF is nutritionally complete, but they were also associated with the fact that children were followed up by a multidisciplinary team during the study period.

Similarly, Orel et al.^16^ found that, after six months of intervention in children with severe neurological impairment and malnutrition, those who were offered CEF presented weight and BMI Z-score, as well as significantly higher percentages of fat and lean mass than those who were offered BTF planned and guided by a nutritionist. The lower effectiveness of BTF was justified by the probable less-than-recommended intake of blenderized feeding. The reduced intake may have occurred as a result of changes made by the caregiver, both in the selection and in the quantities of food. In addition, individuals with severe neurological impairment commonly have slow gastric emptying and gastroesophageal reflux, which results in low tolerance to the large volumes offered in the BTF.

Higher levels of family satisfaction have been demonstrated with the use of BTF in six studies.^19^ ^,^ ^21^ ^,^ ^22^ ^,^ ^25^ ^–^ ^27^ The main positive points mentioned by the families were: being able to provide “real foods,” that is, diets consisting of real foods, reduction of symptoms of intolerance to tube feeding, greater oral intake, having family meals, and not using CEF, which they mentioned to dislike.

A small number of parents did not adhere to the use of BTF claiming lack of knowledge or time constraints.^26^ The main difficulties identified in relation to the use of BTF were longer preparation time, the need for follow-up by a trained professional, potential nutritional inadequacy of the diet, insecurity regarding satisfactory weight gain, difficulty in feeding the child outside home, difficulty in the storage of feedings, and possibility of obstruction of the probes.^25^ ^,^ ^27^

Regarding access to information on the use of BTF, in the study conducted by Trollip et al.^25^ parents reported that access mainly occurred through online searches on social media and support groups. In the same study, a third of parents and/or caregivers mentioned the difficulty in knowing the nutritional composition of foods and expressed the need for knowledge of nutrition and of which foods to use in the diet.

In the investigation by Johnson et al.,^26^ only half (49.3%) of parents received support from healthcare professionals to provide prescriptions and supervise the BTF. The others obtained information from the internet, in printed materials or in face-to-face or online support groups composed of other families that used the BTF. In this context, the authors reinforce that children fed by probes have unique nutritional needs and require individualized nutritional follow-up by a trained healthcare professional.

This integrative review has strengths, as it followed the PRISMA guidelines and relied on independent reviewers to identify and assess the quality of the selected studies. The articles included in the discussion are current, demonstrating the relevance and practical application of the theme. Most of them were cohorts, but the sample size was not very significant. There was a limited number of research that evaluated the nutritional composition of BTF in children. In three studies, information was obtained from data self-reported by telephone, online form, and validated online research instrument, a fact that can generate bias.

The present review enabled to evaluate relevant aspects of BTF in comparison to CEF, such as nutritional composition, health outcomes, and family satisfaction involving children using an alternative feeding route other than the mouth. Most of the included studies demonstrated positive results after the introduction of BTF as a partial or complete support for nutrition. It was found that it is possible to guarantee BTF with complete and satisfactory nutritional composition for children, as long as it is properly planned and guided by a nutritionist or a trained multidisciplinary team. The authors highlight the use of BTF associated with positive health outcomes such as reductions in hospitalizations and in gastrointestinal symptoms. A high frequency of family satisfaction was verified. Conversely, for children who already suffered from malnutrition, BTF was not able to guarantee the same nutritional recovery as the commercial feeding, considering that CEF is more effective and safer for this group of children.

Studies that evaluate the use of BTF in children's nutrition are still scarce and present methodological weaknesses, in such a way it is not possible to establish more robust conclusions. Studies of better quality, longitudinal and case-control types should be conducted with as many participants as possible, allowing to assess the causal relationship between the use of BTF and possible short- and long-term benefits.
